# Calcified or ossified benign soft tissue lesions that may simulate malignancy

**DOI:** 10.1007/s00256-019-03272-3

**Published:** 2019-07-10

**Authors:** Robert M. Kwee, Thomas C. Kwee

**Affiliations:** 1 Department of Radiology, Zuyderland Medical Center, Heerlen/Sittard/Geleen, The Netherlands; 2 Department of Radiology, Nuclear Medicine and Molecular Imaging, University Medical Center Groningen, University of Groningen, Hanzeplein 1, PO Box 30.001, 9700 RB Groningen, The Netherlands

**Keywords:** Soft tissue lesion, Calcifications, Ossification, Benign, Malignant

## Abstract

The purpose of this article is to review calcified or ossified benign soft tissue lesions that may simulate malignancy. We review the clinical presentations, locations, imaging characteristics, and differential diagnostic considerations of myositis ossificans, tophaceous gout, benign vascular lesions, calcific tendinopathy with osseous involvement, periosteal chondroma, primary synovial chondromatosis, Hoffa’s disease, tumoral calcinosis, lipoma with metaplasia, calcifying aponeurotic fibroma, calcific myonecrosis, ancient schwannoma, and Castleman disease.

## Introduction

Many patients are referred to the radiology department for evaluation of a soft tissue lesion in daily practice. The vast majority of soft tissue lesions are benign, with a very good outcome after resection [1]. Malignant soft tissue lesions are rare, but they are potentially life-threatening and may pose a diagnostic and therapeutic challenge [1]. It is the task of the radiologist to differentiate lesions that are certainly benign from those that are not, and to provide a differential diagnosis for lesions that appear malignant or are of unclear nature in terms of benignancy or malignancy on imaging [2]. The identification of calcifications or ossification in a soft tissue lesion is important because it can narrow the differential diagnosis or be suggestive of a certain diagnosis when combined with other imaging findings. In addition, differentiating between calcification and ossification can narrow the differential diagnosis as some pathologies tend to produce ossifications while other pathologies tend to produce calcifications. The purpose of this article is to review calcified or ossified benign soft tissue lesions that may simulate malignancy. We first discuss how soft tissue calcifications can be differentiated from ossification on imaging. Subsequently, we review the clinical presentations, locations, imaging characteristics, and differential diagnostic considerations of several benign entities with calcifications or ossifications that may simulate malignancy.

## Calcifications versus ossification

**Fig. 1:**
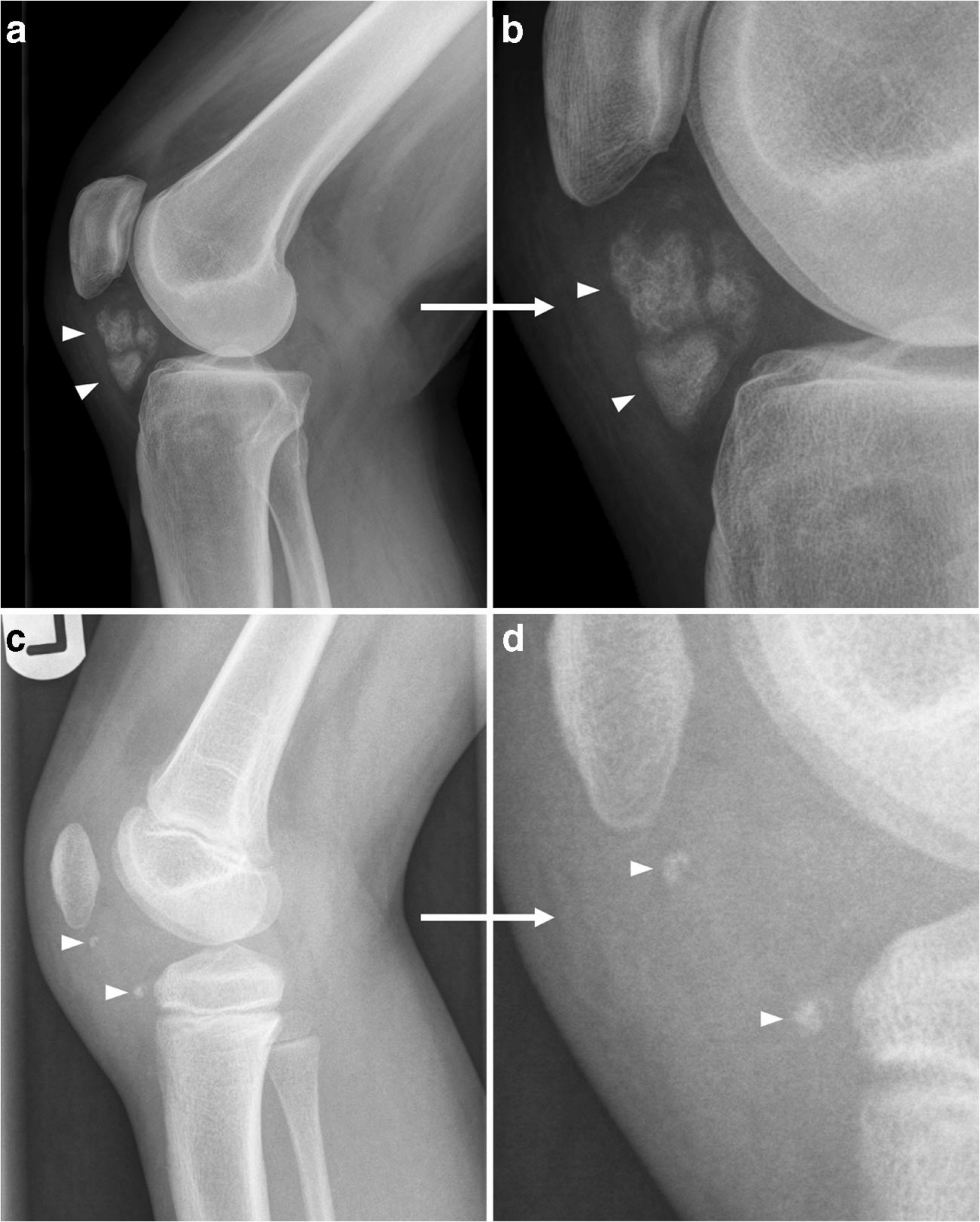
Ossification versus calcification.** a**,** b** Mature bone shows an outer cortex and inner trabecular pattern.** c**,** d** Calcifications normally appear as mineralized densities

Differentiation between calcifications and ossification is very important in making the correct (differential) diagnosis. Radiography is usually the initial imaging modality by which soft tissue calcifications and ossification are detected. Calcifications normally appear as mineralized densities, whereas mature bone shows an outer cortex and inner trabecular pattern (Fig. 1) [3]. CT is more sensitive in detecting calcifications and ossification. Hounsfield unit (HU) values for calcifications show a wide range (usually between 100 and 400), whereas bone reaches higher HU values (700 for trabecular bone and > 1500 HU for cortical bone [3]. On ultrasound, it may be difficult to distinguish between calcifications and ossification, especially when acoustic shadowing is present. Calcifications usually demonstrate uniformly low signal intensity on all MRI sequences. Sensitivity for the detection of small calcifications may be improved by using gradient echo and susceptibility-weighted imaging. Phase images from susceptibility-weighted imaging may also allow for a better distinction between calcifications and other causes of susceptibility variations [3]. Nevertheless, calcifications may still be overlooked on MRI, particularly when small and surrounded by other low-signal-intensity structures such as tendons, ligaments, and fibrosis. Therefore, it is important to correlate MRI with other imaging modalities (including radiography, CT, and ultrasound) when available. Mature bone shows fatty bone marrow within trabecular spaces on both CT and MRI. However, immature bone is not well organized and may be more difficult to discriminate from calcifications [3].

## Myositis ossificans

**Fig. 2:**
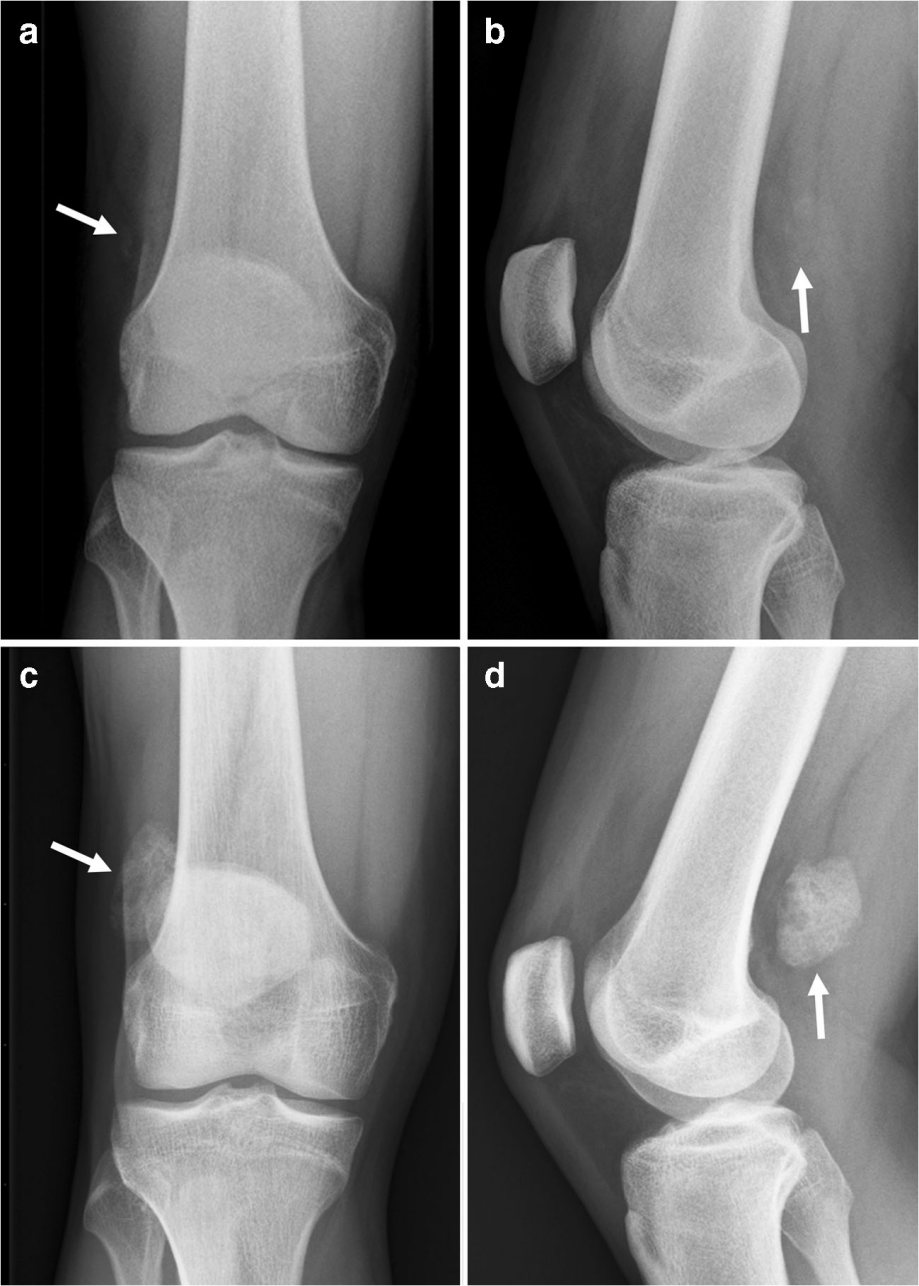
A 24-year-old man with myositis ossificans, early to intermediate stage (**a**,** b**) and late stage (**c**,** d**).** a**,** b** Initial radiography shows amorphous calcifications in the soft tissue at the posterolateral side of the distal femur.** c**,** d** Follow-up radiography after 1.5 years shows ossification

**Fig. 3:**
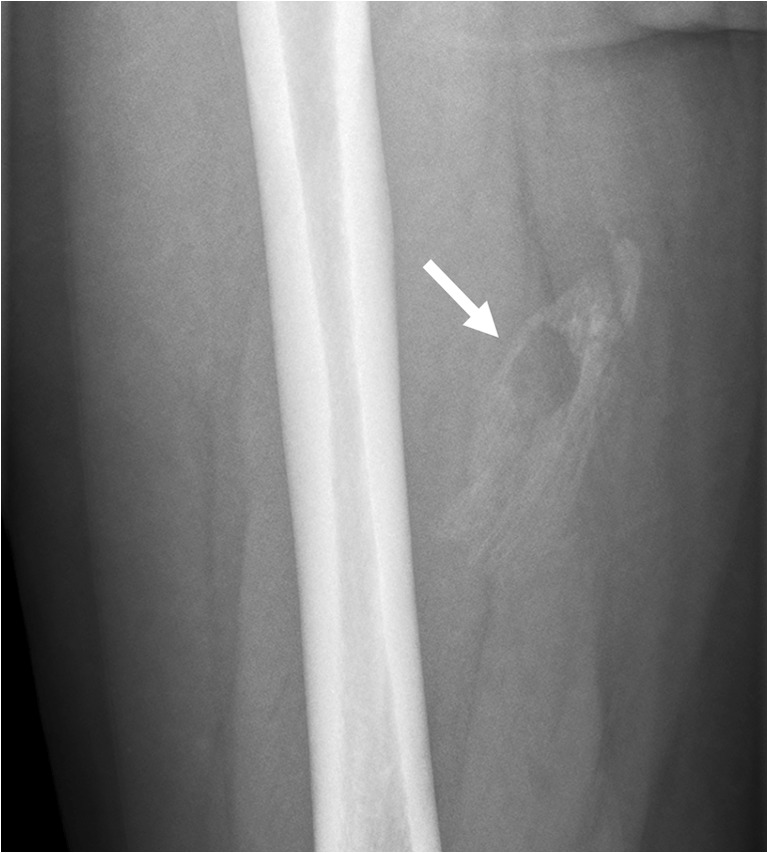
A 54-year-old woman with myositis ossificans, mature stage. Radiography shows a well-defined lesion with peripheral ossification in the soft tissue at the medial side of the right femoral diaphysis (*arrow*), representing mature stage myositis ossificans

**Fig. 4:**
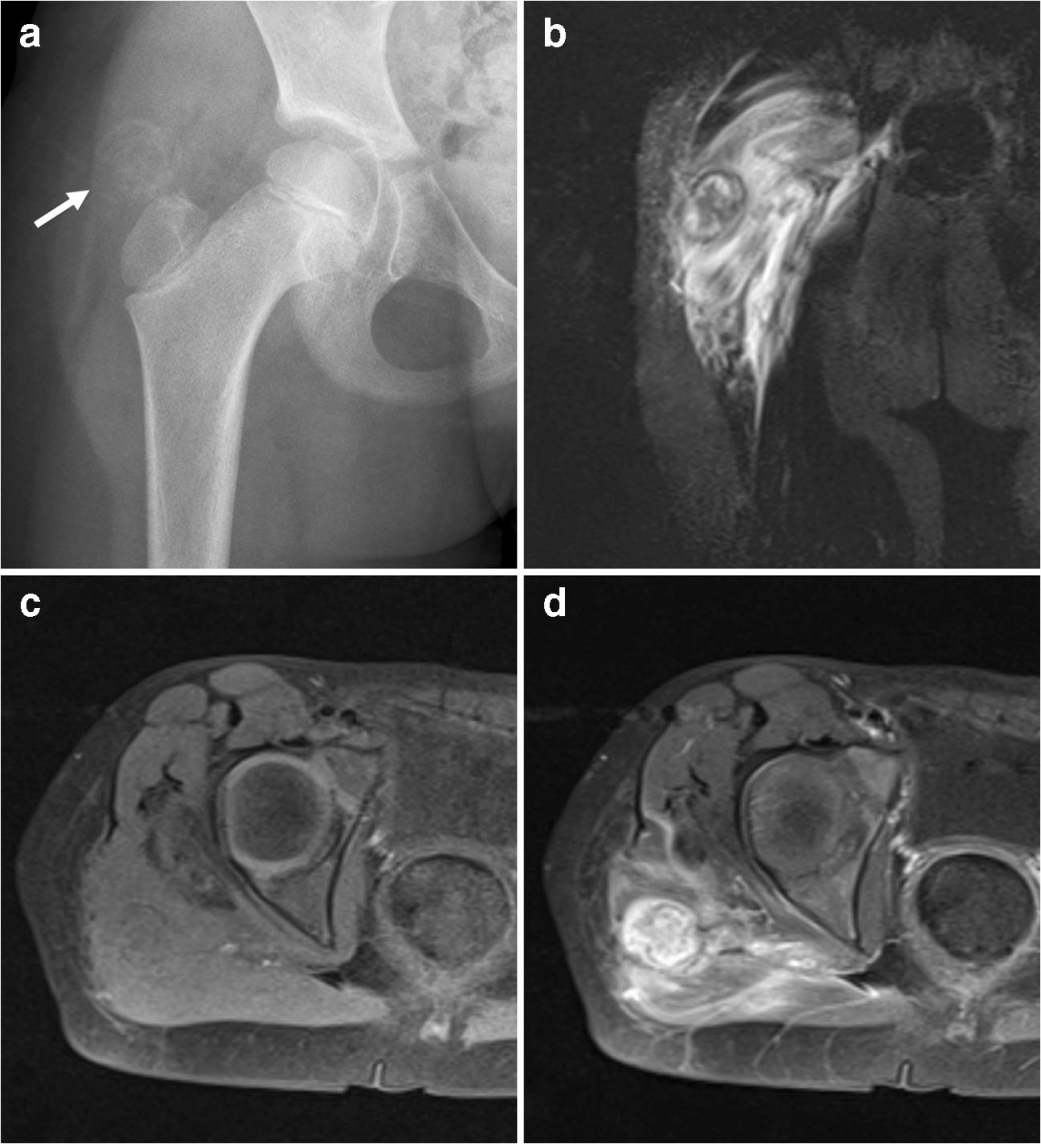
A 6-year-old girl with myositis ossificans, intermediate stage. **a** Radiography shows an ill-defined calcified lesion cranial to the right greater trochanter. **b** Coronal turbo inversion recovery magnitude image shows extensive soft tissue edema surrounding the lesion in the right gluteus maximus muscle.** c**,** d** Coronal pre- (**c**) and postgadolinium (**d**) T1-weighted images with fat suppression show enhancement of the lesion in the right gluteus maximus muscle and surrounding soft tissue. Because of persisting pain, the lesion was finally resected and pathologically proven to be myositis ossificans

Myositis ossificans is a benign ossifying soft tissue mass typically occurring within skeletal muscle [4]. Of note, the term “myositis ossificans” is a misnomer when more broadly used to discuss heterotopic ossification, as heterotopic ossification is neither specific to muscle nor involves prominent inflammation after its early stages [5]. Nevertheless, “myositis ossificans” is still a widely used term, both among pathologists, orthopedic surgeons, and radiologists [4–7], and will be used in this article to refer to this entity. The pathophysiology of myositis ossificans is incompletely understood [6]. The classic clinical history is pain, swelling, and joint stiffness following blunt soft tissue trauma; young active males are most commonly affected [6]. However, clinical presentation is variable and depends on disease stage [6]. Furthermore, a history of trauma is often inapparent [4, 6]. In fact, trauma to the affected region is not required for the development of myositis ossificans. For example, it is also frequently seen around joints in patients with acquired neurologic deficit (also known as neurogenic paraosteoarthropathy). Myositis ossificans progresses through several stages: an early (< 4 weeks), intermediate (4–8 weeks), and mature stage (> 8 weeks) (Fig. 2) [6]. In the early stage, calcifications are usually not apparent. In the intermediate stage, amorphous calcifications appear. These calcifications produce a densely calcified peripheral rim with a lucent center, typically by the end of the intermediate stage. The mature stage is characterized by a peripheral rim of calcification resembling cortical bone (Fig. 3). Mature lesions may show diffuse ossification, typically run in parallel with the long axis of the muscle, and often have a radiolucent cleft that separates it from adjacent bone [6]. Nevertheless, it is important to note that ossification adherent to bone does not exclude myositis ossificans. Notably, the rapidity of the appearance and maturation of the calcification in myositis ossificans is highly dependent on the patient’s age, similar to callus formation. This is because myositis ossificans is basically represents richly vascularized granulation tissue differentiating to bone. Just as callus forms and matures more rapidly in younger patients, the same is true of myositis ossificans. The time frames listed for the various stages are not necessarily accurate because of age dependence. This typical evolution at radiography and CT is diagnostic for myositis ossificans. Myositis ossificans has a varying appearance at MRI, depending on disease stage [4, 6]. In the early and intermediate stages, lesions may be poorly defined and show enhancement, hemorrhage, fluid-fluid levels, and surrounding soft tissue edema (Fig. 4) [4, 6, 7]. In the mature stage, myositis ossificans tends to be well defined with internal trabecular bone with mature fat (corresponding to marrow in heterotopic bone) and resolution of surrounding edema [4, 6, 7]. Similar to enhancement at MRI, myositis ossificans can show intense tracer uptake at bone scintigraphy and FDG PET in early and intermediate stages, which progressively reduces as maturation occurs [6, 8]. Diagnosis of myositis ossificans can be challenging in early and intermediate stages, especially when there is no clear history of trauma. Parosteal osteosarcoma, soft tissue sarcoma associated with calcifications (such as synovial sarcoma), and soft tissue abscess are in the differential diagnosis. Close imaging surveillance or biopsy are warranted in indeterminate cases. However, in the early stage it may also be difficult to pathologically distinguish myositis ossificans from extraskeletal osteosarcoma [6]. When in doubt about the diagnosis of myositis ossificans, patients should be referred to a tertiary care center with expertise in sarcomas.

## Tophaceous gout

**Fig. 5:**
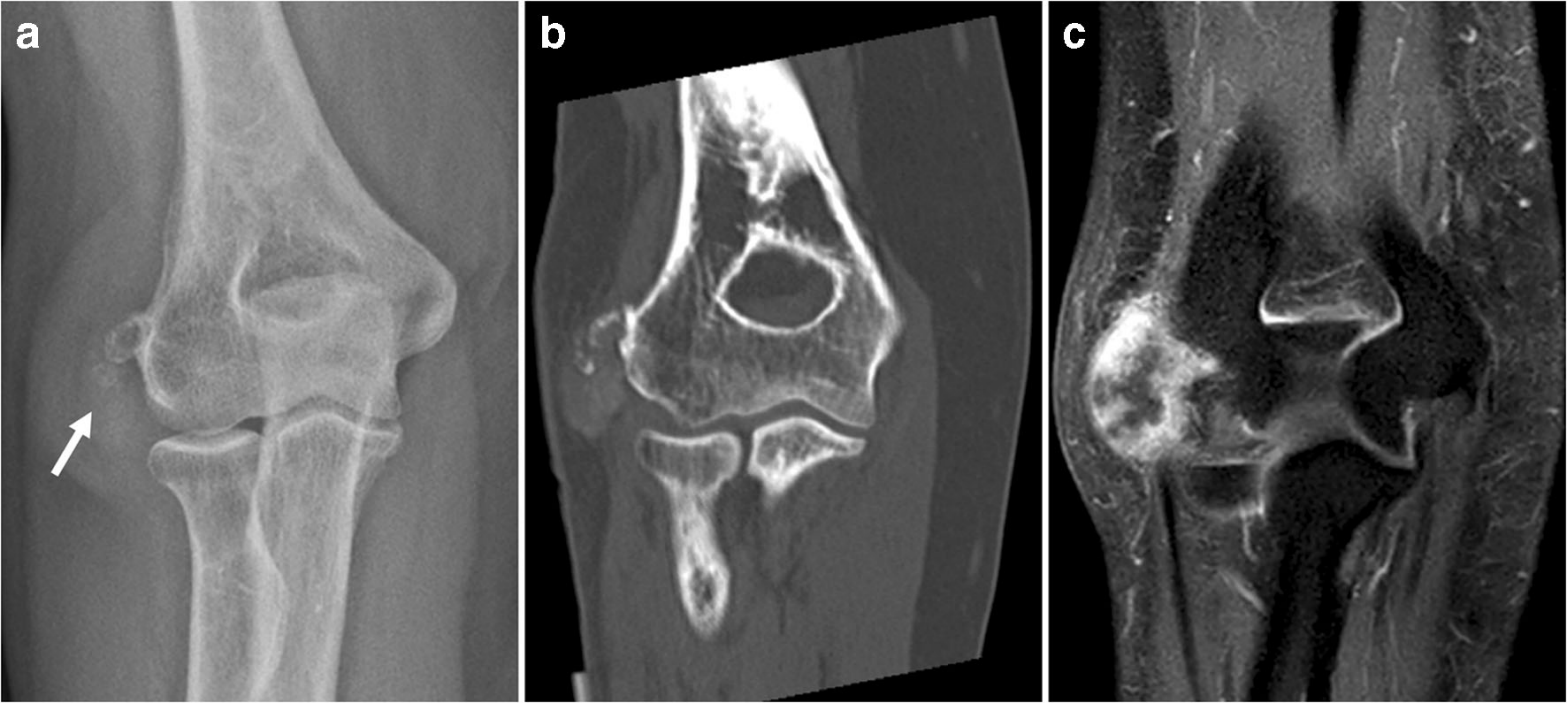
A 60-year-old woman with tophaceous gout.** a**,** b** Radiography (**a**) and coronal CT (**b**) show a soft tissue lesion with calcifications near the lateral epicondyle of the distal humerus. **c** Coronal postgadolinium T1-weighted images with fat suppression demonstrates peripheral enhancement of the lesion. Biopsy was performed, demonstrating monosodium urate crystals, consistent with gout

Gout is a crystal arthropathy resulting from monosodium urate crystal deposition in and around the joints. Monosodium urate crystals can precipitate with calcium when extraarticular and not in solution (intraarticular). Transplant recipients, females, and elderly people in particular can develop gouty tophi as an initial manifestation of their disease [9]. Tophaceous gout can affect almost any part of the body and mimic a malignant soft tissue tumor [9, 10]. At radiography and CT, hyperdense (Fig. 5) soft tissue swelling can be seen and presence of dense calcifications is a late finding [11]. At ultrasound, tophi are generally hyperechoic, heterogeneous, poorly defined, multiple grouped, and surrounded by an anechoic halo [12]. At MRI, they generally show intermediate T1 signal intensity, variable T2 signal intensity, and enhancement (Fig. 5) [13]. These MRI features do not help to discriminate tophaceous gout from a malignancy (e.g., synovial sarcoma), especially when the clinical and biochemical presentation and location are atypical. However, multifocality is often a clue that a malignant neoplasm is unlikely. Biopsy may be needed to definitively establish the diagnosis. Alternatively, dual-energy CT may be used, as it can accurately identify monosodium urate crystals [14, 15]. It is important to mention that biopsy of gout can be very confusing and difficult for pathologists for diagnosis if the material is placed in typical formalin as the diagnostic needle-shaped crystals dissolve. An alcohol solution should be used if gout is a potential diagnosis at biopsy, or the biopsy sample should be sent unfixed or “fresh”.

## Benign vascular lesions

**Fig. 6:**
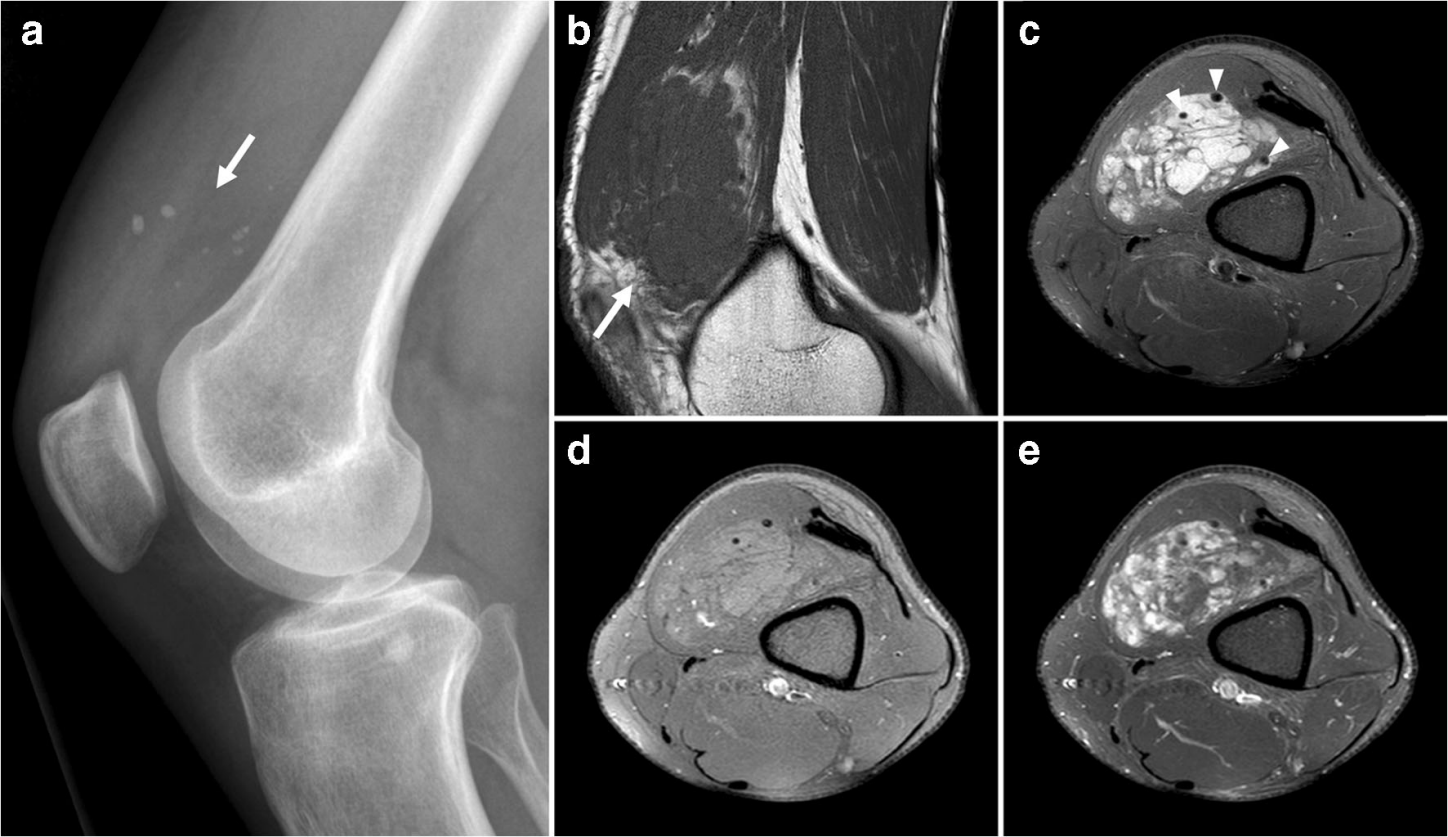
A 38-year-old man with a venous malformation.** a** Radiography shows suprapatellar phleboliths.** b** Sagittal T1-weighted image shows a lobulated mass with low signal intensity.** c** Axial proton-density weighted image with fat suppression shows the mass in the vastus medialis muscle to have high signal intensity and internal signal voids (*arrowheads*), representing phleboliths.** d**,** e** Axial pre- (**d**) and postgadolinium (**e**) T1-weighted images with fat suppression show enhancement of the mass. In this figure, fat overgrowth and serpentine morphology are also keys to the correct diagnosis of venous malformation

Venous malformations are the most common peripheral vascular malformation. They can occur anywhere, but are usually located in the head and neck (40% of cases), trunk (20%), and extremities (40%) [16]. Venous malformations increase proportionally with body growth without regression [16]. Patients usually become symptomatic in late childhood or early adulthood [16]. Venous malformations may permeate across tissue planes and invade multiple adjacent tissues (fat, muscle, tendon, bone) [16]. When they are superficially located, venous malformations appear as faint blue, soft, easily compressible, and nonpulsatile masses [16]. They characteristically enlarge with the Valsalva maneuver and in dependent positions [16]. Symptoms may include pain, impaired mobility, and skeletal deformity [16]. Phleboliths are present in approximately 50% of venous malformations (Fig. 6) [17] and are the result of stagnant blood flow due to thrombosis [18]. CT is more sensitive than radiography in detecting phleboliths [17]. Unlike slow-flow vascular malformations (both venous malformations and hemangioma) that tend to produce phleboliths, high-flow vascular lesions tend to produce more “tram-track” calcifications seen in arteries. Vascular malformations have a variable ultrasound appearance: they can be ill defined or well defined, typically have a heterogeneous echogenicity with multiple cystic spaces, and they may or may not show vascularity on color Doppler [19]. Nevertheless, these lesions frequently exhibit a typical “sponge”-like appearance on ultrasound and can be compressed with mechanical pressure. The standard for imaging evaluation is MRI (Fig. 6). Venous malformations appear as a septated lobulated mass without mass effect and usually demonstrate (besides phleboliths) fluid-fluid levels, low T1 signal intensity, high T2 signal intensity, no-flow voids, and slow gradual enhancement on delayed images [16]. Fat is commonly interspersed between stromal elements and fluid pockets, and there are commonly tubular structures (serpentine morphology). Perilesional edema may be present [16]. Given that imaging is often diagnostic, biopsy can generally be avoided [17]. Notably, biopsy attempts may yield insufficient solid tissue for pathologic analysis and can lead to bleeding complications [17].

## Calcium hydroxyapatite deposition disease

**Fig. 7:**
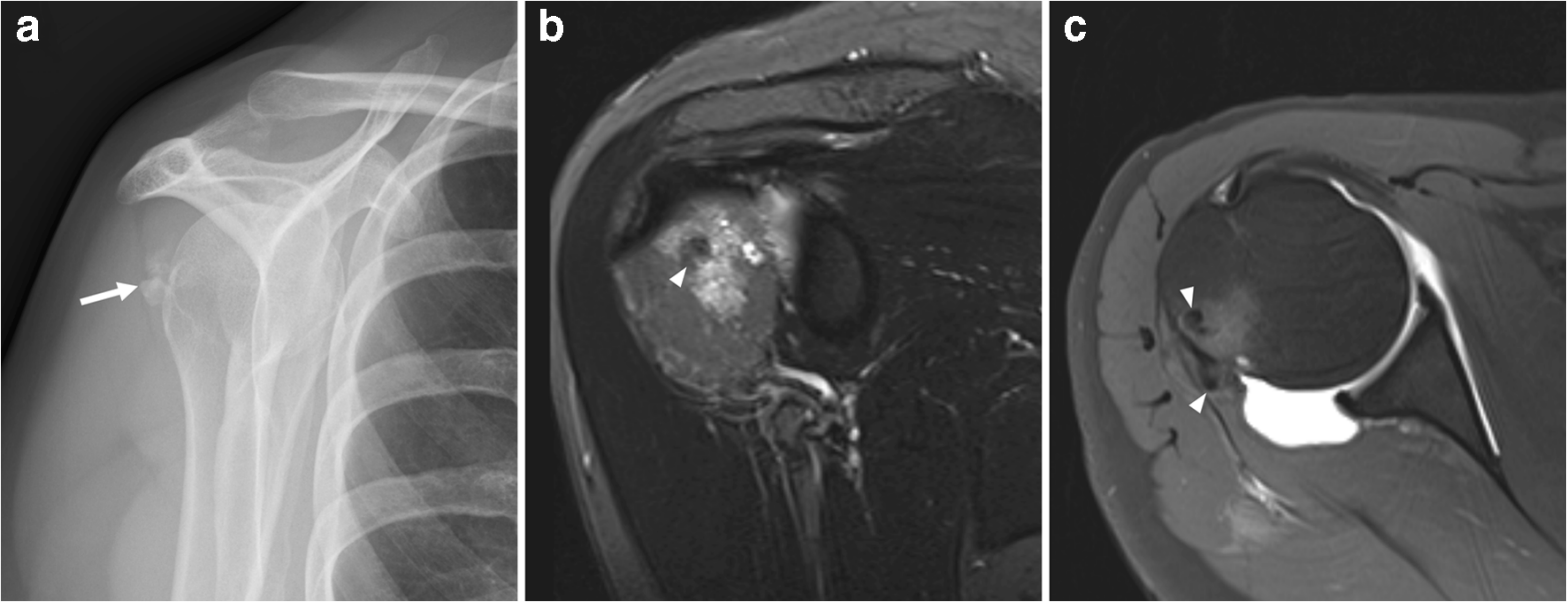
A 53-year-old woman with calcific tendinopathy of the infraspinatus tendon with osseous involvement.** a** Radiography shows soft tissue calcifications posteriorly to the humeral head.** b** Coronal oblique turbo inversion recovery magnitude image shows intra-osseous extension of calcification (*arrowhead*) and surrounding bone marrow edema. **c** Axial T1-weighted image with fat suppression after intra-articular injection of gadolinium shows calcifications which are located in the infraspinatus tendon and in the humeral head (*arrowheads*). There is no associated soft tissue mass

Calcific tendinopathy is a common, usually self-limiting disorder due to deposition of calcium hydroxyapatite within tendons, usually of the rotator cuff. The adjacent bone may occasionally be involved [20–23], which most commonly occurs in the femur and humerus (typically the humeral and femoral tuberosities) [20]. Other soft tissue structures like bursae and joint capsule may also be involved. Pain is frequent, although patients may be asymptomatic [20]. Calcific tendinopathy with osseous involvement has an aggressive imaging appearance. Marked edema and enhancement in the bone around the calcifications (which is the main finding), cortical destruction, and periosteal reaction may mimic malignancy (e.g., soft tissue sarcoma, periosteal sarcoma, or metastatic disease) or infection (Fig. 7) [20–23]. Correlating MRI with radiographic findings or CT is important because tendon calcifications might not be readily appreciated on MRI alone [20, 22]. The characteristic location of calcifications in a tendon and absence of an associated soft tissue mass should provide the necessary clues to the correct diagnosis and avoidance of biopsy [20–23]. The other feature that is helpful to distinguish calcific tendinopathy from malignancy is the prominent associated bone marrow edema on MRI, which is not a typical finding with deep-seated sarcomas. Importantly, calcium hydroxyapatite deposition disease does not have to occur within the tendon, and it occurs in many places besides the rotator cuff. It is frequently seen around the hip and is particularly difficult to distinguish from tumor/infection/trauma when it occurs in the cervical spine. Therefore, this diagnosis should always be considered in the setting of acute onset pain and periarticular calcifications.

## Periosteal chondroma

**Fig. 8:**
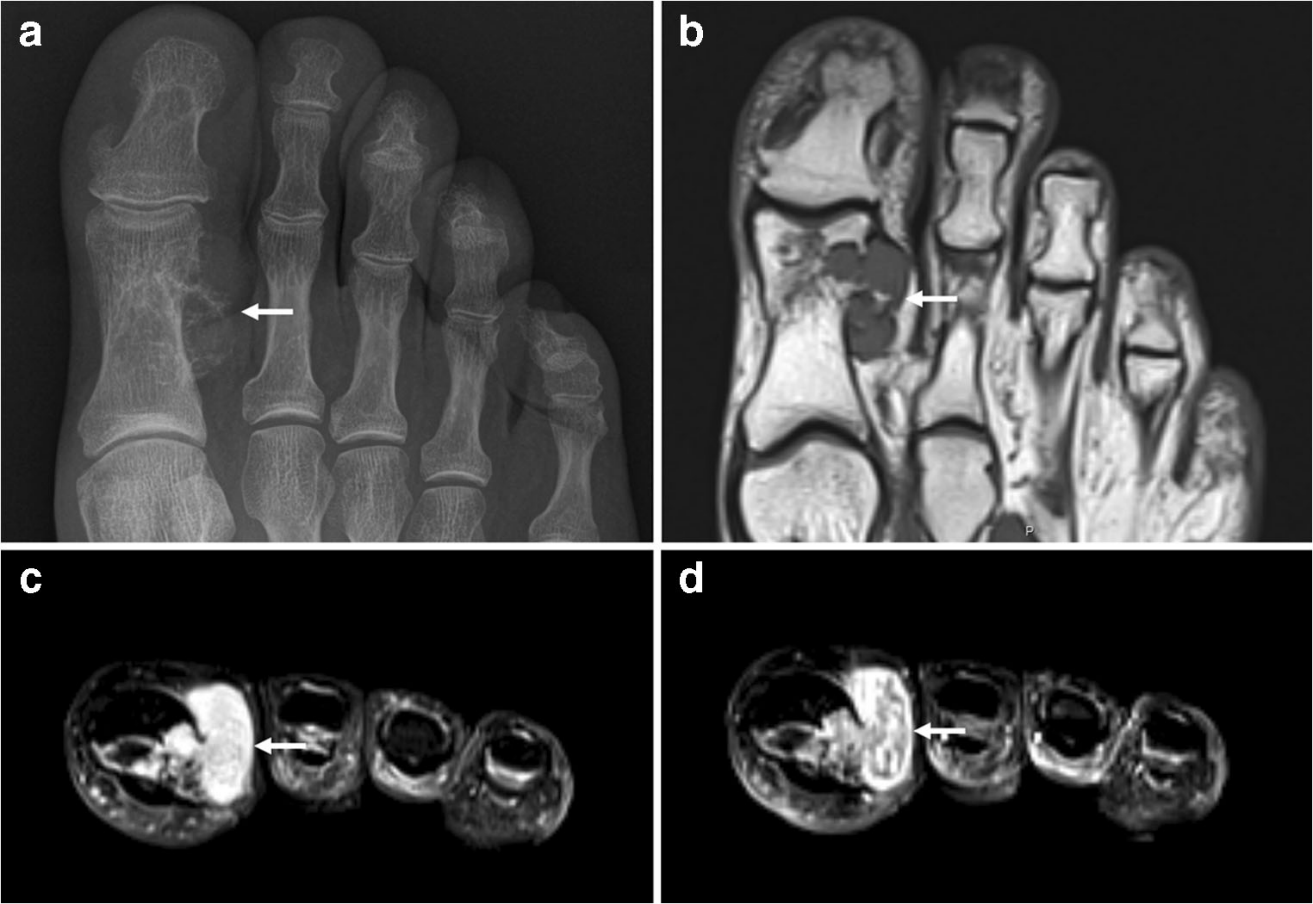
A 48-year-old man with periosteal chondroma.** a** Radiography shows soft tissue lesion with a calcified chondroid matrix lateral to the proximal phalanx of the first digit.** b**–**d** Axial T1-weighted image (**b**), coronal proton-density-weighted image with fat suppression (**c**), and coronal postgadolinium T1-weighted image with fat suppression (**d**) show pressure erosion of neighbouring bone without associated medullary bone. The lesion also displaces the flexor hallucis longus tendon. There is no soft tissue edema. The lesion shows a septal enhancement pattern (**d**)

**Fig. 9:**
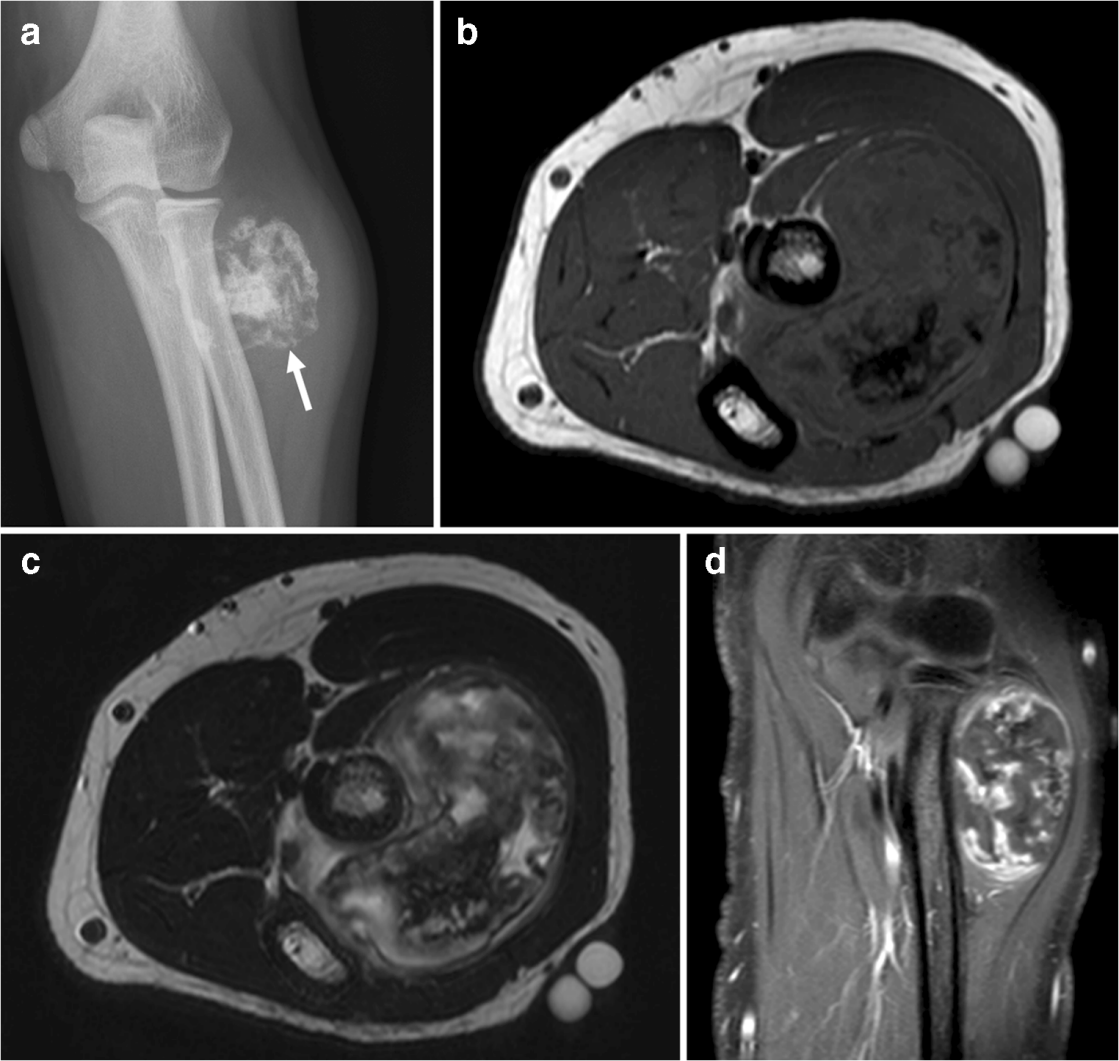
A 16-year-old boy with periosteal chondroma. **a** Radiography shows a calcified chondroid matrix laterally to the proximal radial diaphysis. **b, c** Axial T1-weighted (**a**) and T2-weighted (**b**) images show that the lesion is located directly adjacent to the radius. Signal intensity of the lesion is nearly isointense to muscle on T1-weighted images and hyperintense to T2-weighted images, with areas of signal loss which represent the calcified chondroid matrix. **d** Coronal postgadolinium T1-weighted image with fat suppression shows a septal enhancement pattern

Periosteal chondroma, also known as juxtacortical chondroma, is a rare benign chondral tumor arising from the periosteum [24, 25]. Preferred locations are metaphyses of long tubular bones, hands and feet, and the osseous insertions of tendons and ligaments [24]. It predominantly occurs in men who are in their 2nd to 4th decades [24, 25]. Focal swelling and pain are the most common clinical symptoms [24, 25]. The typical radiographic appearance of periosteal chondroma is a calcified chondroid matrix with scalloping and slightly overhanging edges of the cortex of adjacent bone (Figs. 8 and 9) [26]. At MRI, the lesion typically shows a lobulated configuration and no edema in adjacent bone and soft tissue (Figs. 8 and 9). Signal intensity of cartilaginous tissue is T1 hypo- or isointense relative to muscle and T2 hyperintense relative to fat. The calcified chondroid matrix shows signal loss (Figs. 8 and 9). Contrast enhancement is typically according to a “septal pattern”, meaning peripheral enhancement with linear or curvilinear internal enhancement (Figs. 8 and 9) [24]. Imaging features of periosteal chondroma and periosteal chondrosarcoma also show variable overlap [25, 27]. Size is the most reliable indicator: when lesion size exceeds 3 cm, chondrosarcoma is more likely [25]. In addition, periosteal chondroma tends to lack associated medullary bone or soft tissue edema. Nevertheless, chondrosarcomas do not necessarily show edema. Treatment of both lesions is surgical, with a local excision for periosteal chondroma and a wide excision for periosteal chondrosarcoma [25]. Periosteal chondroma may be easier to distinguish from periosteal/parosteal osteosarcoma because the latter shows an osseous rather than a chondroid matrix.

## Primary synovial chondromatosis

**Fig. 10:**
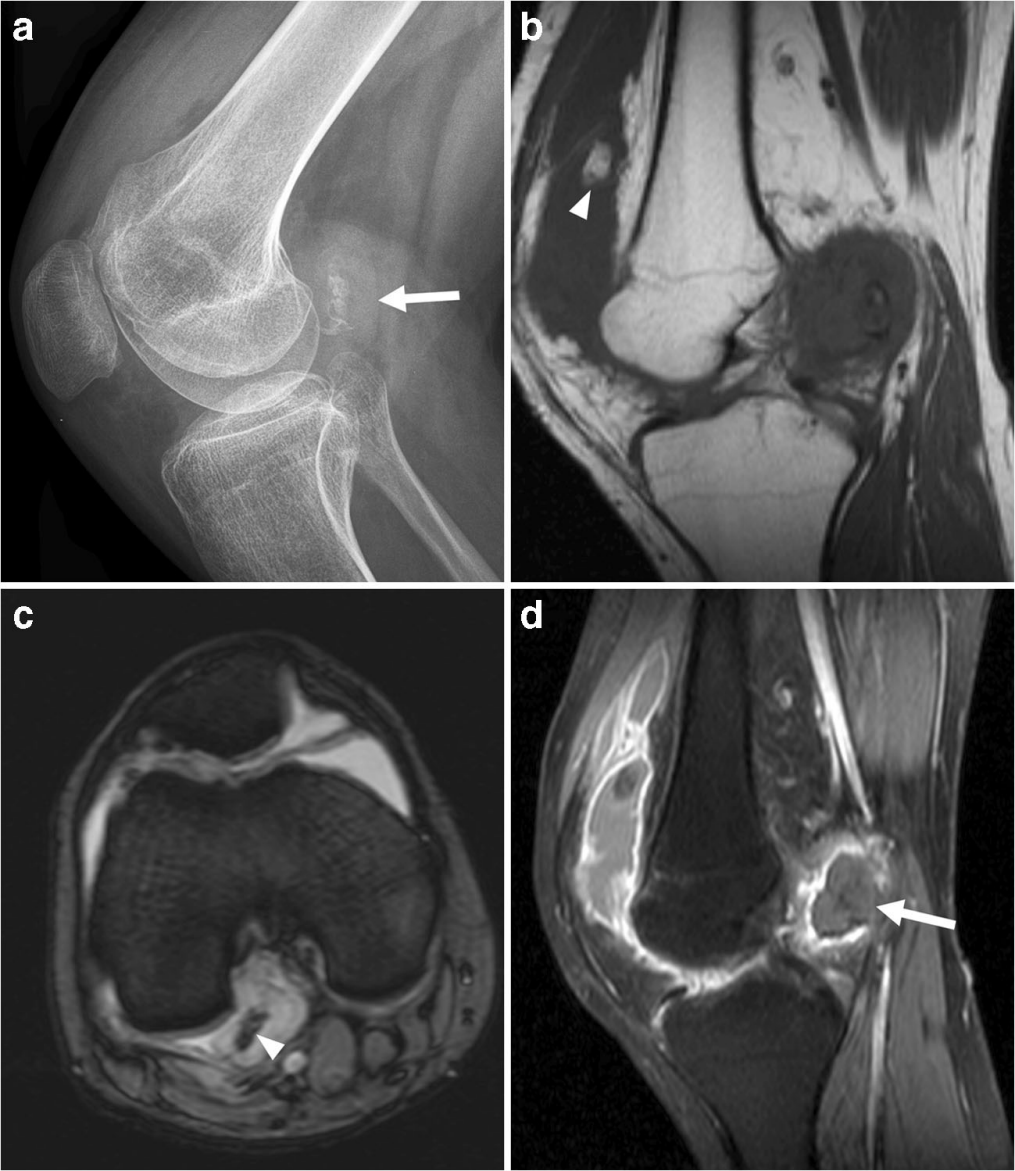
A 19-year-old man with primary synovial chondromatosis of the knee. **a** Radiography shows a soft tissue lesion with ring- and arc-like calcifications posteriorly to the knee. **b** Sagittal T1-weighted image shows an additional ossified fragment in the suprapatellar recess (*arrowhead*).** c** Axial T2*-weighted image shows the calcified part of the lesion as an area of signal loss (*arrowhead*). **d** Sagittal postgadolinium T1-weighted image with fat suppression shows diffusely thickened and enhancing synovium. The lesion posteriorly to the knee (*arrowhead*) shows no internal enhancement

Primary synovial chondromatosis is an uncommon benign neoplastic process with development of intrasynovial cartilage nodules in a joint, tendon sheath, or bursa [28]. These nodules enlarge and detach from the synovium. The disorder most commonly occurs in male adults. The knee and hip are predilection sites [28]. Clinical symptoms typically are pain, swelling, and restriction of range of motion of the affected joint [28]. Radiographs reveal multiple intraarticular calcifications in the majority of cases, which frequently show a pathognomonic appearance of being innumerable and similarly shaped [28]. In addition, a typical chondroid ring-and-arc mineralization pattern may be present (Fig. 10) [28]. However, because the lesions are small, ring-and-arc calcifications are not frequently visible in primary synovial chondromatosis. Fragments may ossify and display a peripheral rim of cortex and inner trabecular bone (Fig. 10) [28]. A target appearance may also be seen, consisting of a single peripheral rim and central dot of calcification [28]. Mechanical pressure of the intra-articular fragments can cause extrinsic bone erosions and secondary osteoarthritis can occur [28]. CT is the optimal imaging modality to evaluate calcifications and detect bony erosions [28]. MRI signal characteristics are variable, depending on the degree of calcifications [28]. Enhancement is typical of hyaline cartilage neoplasms, with a characteristic peripheral and septal pattern [28]. Although uncommon (in approximately 5% of cases), primary chondromatosis can undergo malignant transformation to synovial chondrosarcoma [28, 29]. It can be very difficult to distinguish these entities, both pathologically and radiologically [28, 29]. However, multiple recurrences and development of marrow invasion should be considered alarming features [28].

## Hoffa’s disease

**Fig. 11:**
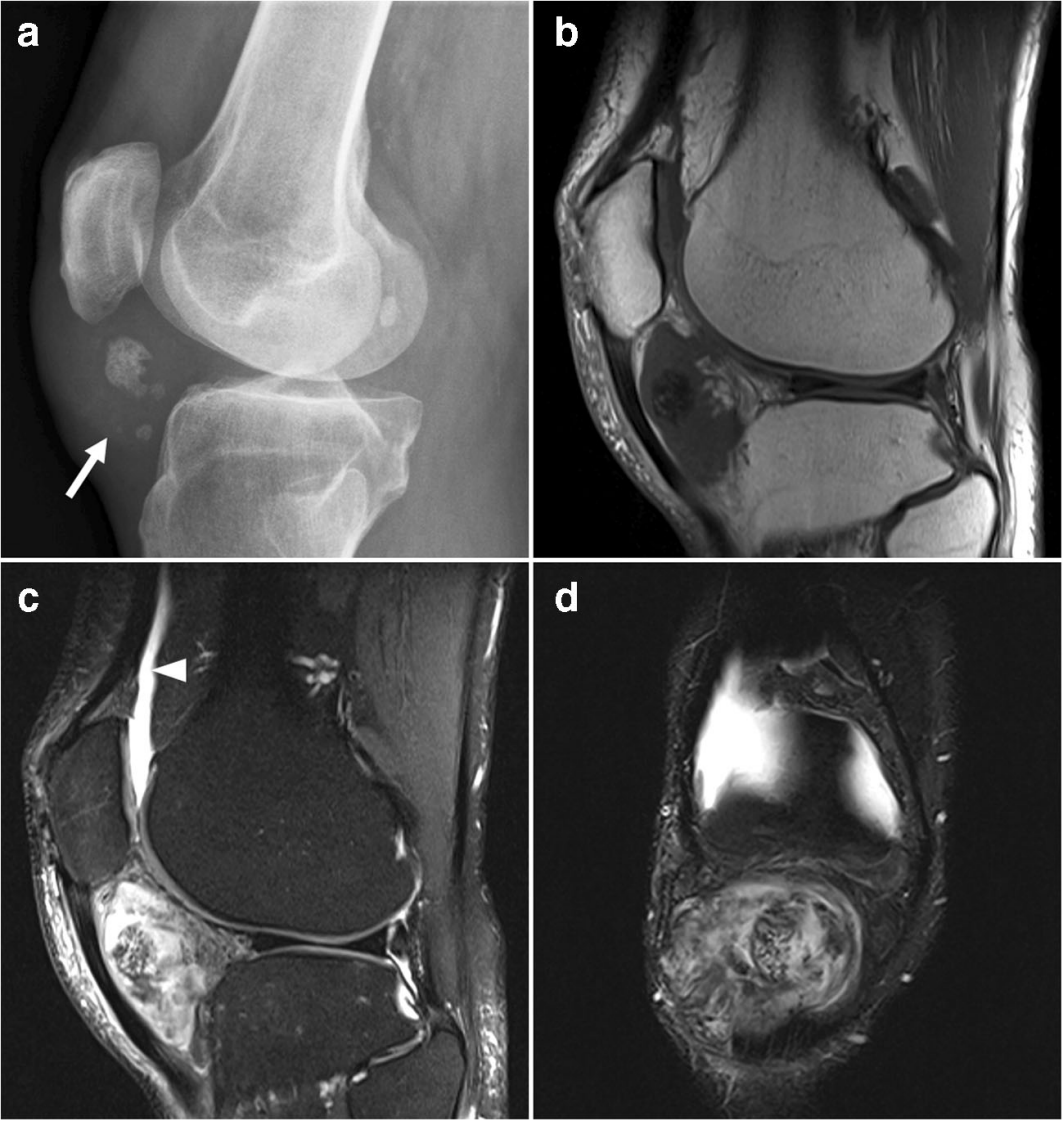
A 68-year-old man with Hoffa’s disease. **a** Radiography shows multiple ossified fragments (arrow) in Hoffa’s fat pad. **b** Sagittal T1-weighted image shows the largest ossified fragment in Hoffa’s fat pad, with surrounding low signal intensity. **c, d** Sagittal (**a**) and coronal (**b**) proton-density-weighted images with fat suppression show high signal intensity in Hoffa’s fat pad with areas of low signal intensity, corresponding to fibrin and hemosiderin

Hoffa disease is a syndrome of impingement and probably caused by acute or repetitive trauma leading to hemorrhage in Hoffa’s fat pad [30, 31]. The inflamed fat pad then becomes hypertrophied, which predisposes it to crushing between the femur and tibia and to a vicious cycle of hemorrhage, inflammation, and further hypertrophy [30, 31]. Hoffa’s disease most commonly occurs in young women, who typically present with anterior knee pain [30]. Practicing of jumping sports and ligamentous laxity causing knee hyperextension are predisposing factors [29]. Acute edema and hemorrhage within the swollen fat pad characteristically manifest as areas of increased T2 signal intensity [30, 31]. Bowing of the patellar tendon from mass effect is seen frequently and a small joint effusion may be present (Fig. 11) [30, 31]. In the subacute and chronic phases, fibrin and hemosiderin have low T1 and T2 signal intensity (Fig. 11) [30, 31]. Fibrous tissue may transform into fibrocartilaginous tissue and eventually ossify [30, 31], which can be seen at radiography (Fig. 11). The exact frequency of ossification in Hoffa’s disease is currently unknown. Synovial sarcoma can arise in Hoffa’s fat pad [32] and could be included in the differential diagnosis, along with venous malformation. However, ossification is an unusual phenomenon in both synovial sarcoma and venous malformation. Therefore, it is very important to differentiate calcifications (which can be present in synovial sarcoma and venous malformation) from ossification at imaging.

## Tumoral calcinosis

**Fig. 12:**
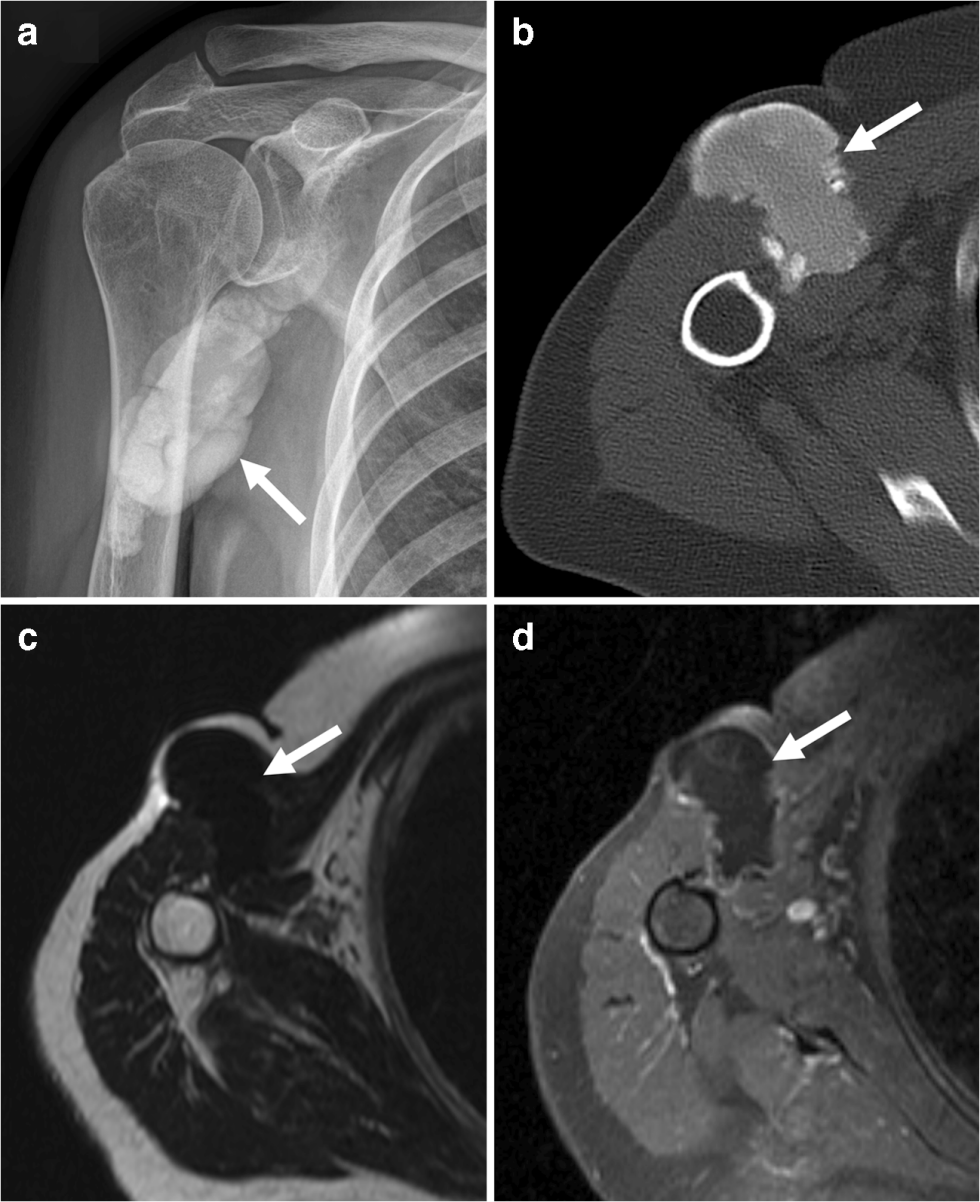
A 62-year-old woman with tumoral calcinosis. **a** Radiography shows a well-circumscribed calcified soft tissue mass (*arrow*) near the right shoulder. **b** Axial CT that the calcified mass is located within the lateral part of the pectoralis major muscle and extends subcutaneously. **c** Axial T2-weighted image shows that the lesion has low signal intensity. **d** Axial T1-weighted image with fat suppression demonstrates low signal intensity of the mass and a thin rim of peripheral enhancement

Tumoral calcinosis is a rare hereditary disease caused by metabolic dysfunction of phosphate regulation associated with massive periarticular calcinosis [33]. Black patients are predominantly affected [33]. Calcified masses most commonly occur around the hip, elbow, shoulder, foot, and wrist, and are not painful unless impinging on a local nerve [33]. Chalky white matter may extrude from late-stage skin ulcers [33]. The radiographic appearance of tumoral calcinosis is a lobulated calcific soft tissue mass (Fig. 12), which is typically cystic, has a bursal distribution, and usually affects extensor surfaces [33]. CT is the modality of choice to delineate the calcific mass (Fig. 12). Multiple fluid-calcium levels may be seen and although there is usually no osseous involvement [33], it is definitely possible for tumoral calcinosis to erode adjacent bone. On MRI, tumoral calcinosis has been reported to appear as a heterogeneous lesion with low T1 signal intensity and high T2 signal intensity [33]. However, in our experience, lesions usually show low T2 signal intensity as would be expected for calcification (Fig. 12). Enhancement may be present in the walls of the cystic parts of the lesion because of hypervascularity [34]. Positron emission tomography is not useful to differentiate tumoral calcinosis from malignancy because tumoral calcinosis can demonstrate avid FDG uptake [35–37]. Differential diagnostic considerations include, amongst others, calcinosis associated with chronic renal failure and soft tissue sarcoma [32]. Careful review of clinical history, laboratory test results, location, and imaging appearance helps to differentiate tumoral calcinosis from these entities. Tumoral calcinosis does not have noncalcified components as expected for synovial sarcoma, extraskeletal osteosarcoma, or chondrosarcoma. Another helpful feature in distinction from malignant neoplasms is the multifocality in most cases of tumoral calcinosis. There are several mimics of tumor calcinosis that share the same radiologic features, including similar distribution, size, and morphology. Mimics of tumor calcinosis can broadly be divided into metabolic calcification (associated with abnormal calcium and/or phosphate levels) and dystrophic calcification (resulting from an underlying inflammatory process, including connective tissue diseases and (atypical) infections), as described in detail elsewhere [33]. The most frequent cause of a periarticular calcified mass is chronic renal failure [33]. Of interest, the specific cause underlying the development of massive periarticular calcification in chronic renal failure remains unknown [33]. Notably, tumoral calcinosis and calcinosis associated with chronic renal failure have similar radiologic and pathologic appearance but treatment is different: surgical excision may be effective in some cases of tumoral calcinosis [33, 38], whereas a more conservative approach is indicated in calcinosis associated with chronic renal failure [38]. Therefore, whenever the diagnosis of tumoral calcinosis is suggested by imaging, calcium-phosphate metabolic parameters and renal function should be checked [38].

## Lipoma with metaplasia

**Fig. 13:**
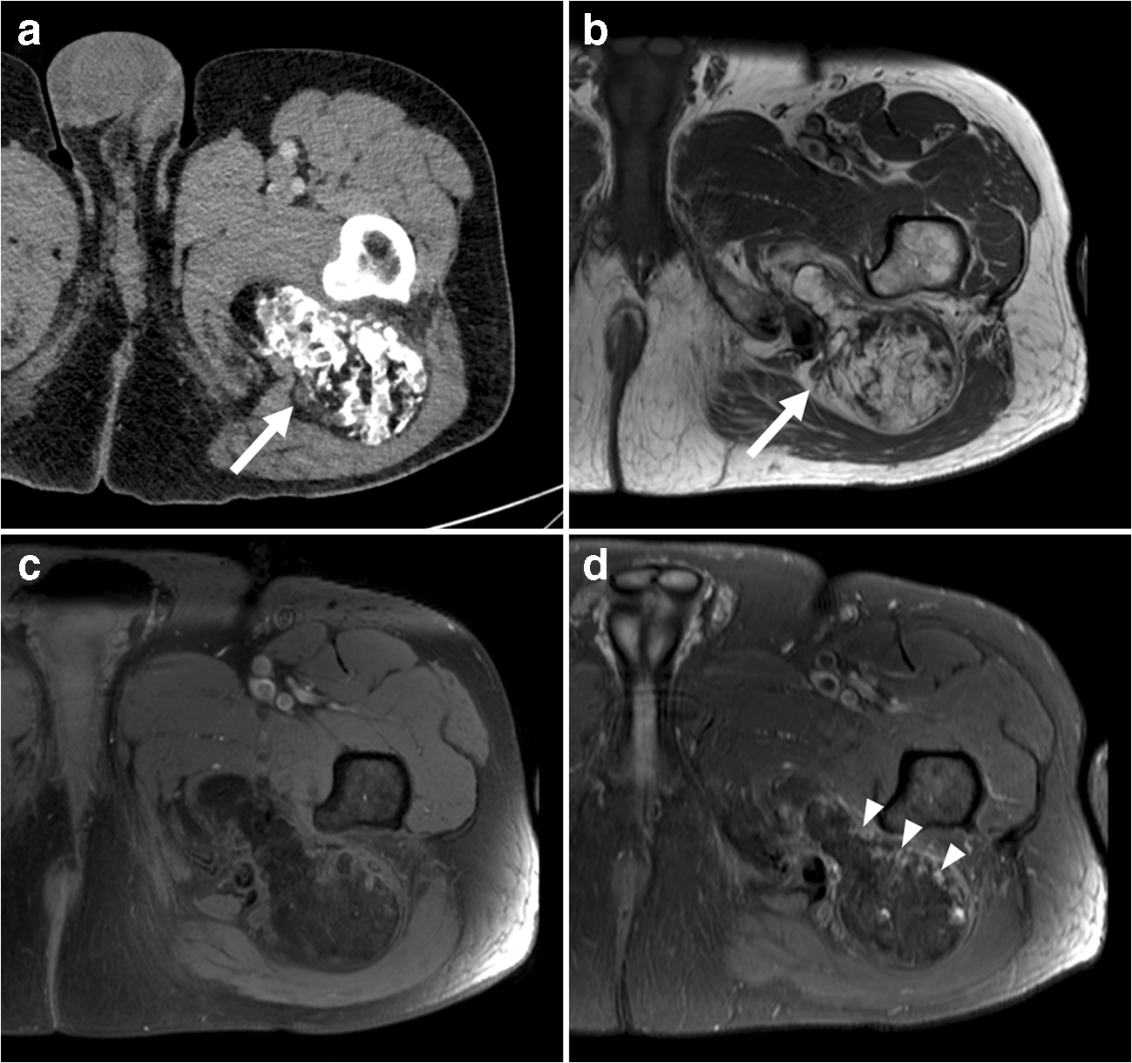
A 68-year-old man with lipoma with metaplastic bone. **a** Axial CT shows a well-defined mass (*arrow*) with ossifications intermixed with fatty tissue in the left ischiofemoral space. **b** Axial T1-weighted image shows the fatty tissue within the mass. **c, d ** Axial pre- (**c**) and postgadolinium (**d**) T1-weighted images with fat suppression show enhancing areas in the anterior part of the mass (*arrowheads*). Pathological analysis after resection confirmed lipoma with metaplastic bone

Lipoma with metaplastic bone (also referred to as osteolipoma) is a rare variant of lipoma in which both mature adipose tissue and mature lamellar bone are present [1, 39, 40]. Patients usually present with a soft tissue mass, which may be painful [39–44]. Lipoma with metaplastic bone may arise in several anatomic locations, including the soft tissues in the neck [43], the oral cavity [42], and extremities [39–41]. Notably, it is considered a different entity than parosteal lipoma, which is also a lipoma variant but contiguous with periosteal bone and commonly associated with reactive changes in the underlying cortex [44, 45]. Prognosis of lipoma with metaplastic bone is similar to simple lipoma and no recurrences after surgical excision have been reported in the literature, to our knowledge [39, 42–44]. On imaging, lipoma with metaplastic bone appears as a well-defined tumor, which respects anatomical boundaries. Besides fat and bone, calcifications can also be present. Radiography can show the calcifications and trabecular bone. When they are largely ossified, lipomas with metaplastic bone may be difficult to appreciate on ultrasound. CT and MRI are the imaging modalities of choice to characterize the tumor and to exactly demonstrate tumor size, location, and extent (Fig. 13). Intratumoral non-fatty components such as bone and cartilage (note that lipomas can also exhibit chondroid matrix calcification and not just ossification) may confound the imaging diagnosis and mimic chondroma or extraskeletal osteochondroma, another rare, benign soft tissue tumor [46]. Lipomas with metaplastic bone may show intense and heterogeneous enhancement (Fig. 13) [39, 41]. In these cases, the possibility of a soft tissue sarcoma (particularly liposarcoma with calcifications and ossification [47]) should be included in the differential diagnosis and pathological evaluation is warranted. Similar to the radiologist, the pathologist may also be confused in making the correct diagnosis because of the presence of non-fatty components within the tumor. Close correlation between imaging and pathological findings aids in correctly diagnosing lipoma with metaplastic bone. Cytogenetic analysis may be used in larger tumors with worrisome features [39].

## Calcifying aponeurotic fibroma

**Fig. 14:**
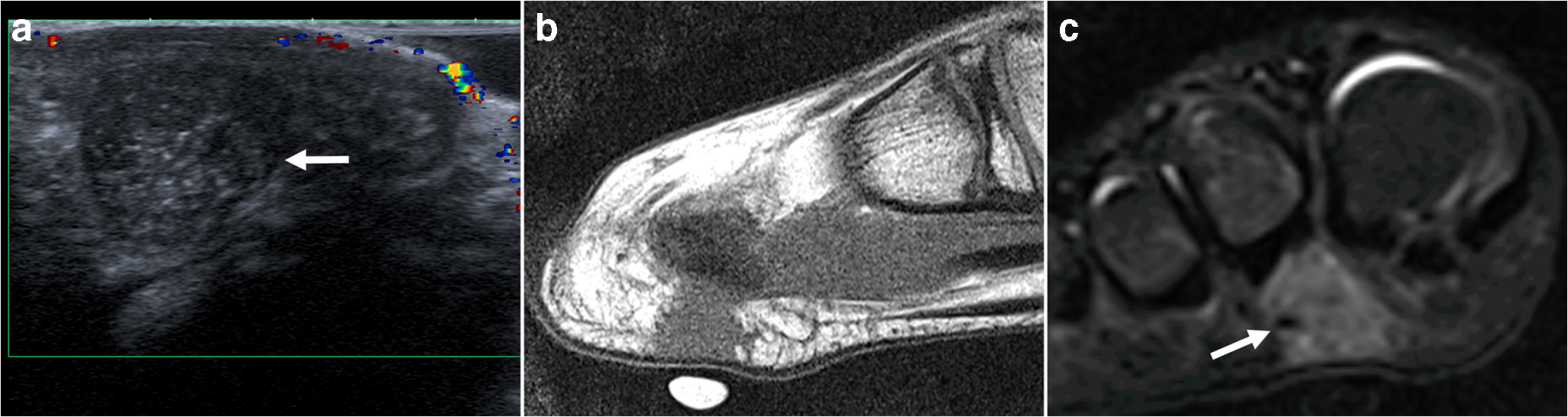
A 13-year-old boy with calcifying aponeurotic fibroma. **a** Ultrasound image in the sagittal plane shows a well-defined hypoechoic mass (*arrow*) with multiple fine hyperechoic reflections (representing fine calcifications) at the plantar side of the right forefoot. **b** Sagittal T1-weighted image demonstrates the mass (near the skin marker) to be isointense to muscle. **c** Coronal turbo inversion recovery magnitude image demonstrates the mass (*arrow*) to be hyperintense. Pathological analysis after resection confirmed aponeurotic fibroma with presence of multiple fine calcifications

Calcifying aponeurotic fibroma is a rare benign but locally aggressive fibroblastic tumor that most commonly affects the deep volar fascia, tendons, and aponeuroses of the hands, followed by the feet [48]. Less frequently involved sites include the neck, thigh, forearm, popliteal fossa, and lumbosacral region [48]. It typically occurs in children and adolescents, and males are more commonly affected than females [48]. It is one of the few fibrous lesions that can calcify [48]. Therefore, it should be included in the differential diagnosis when a calcifying mass is seen in young children around the hand/wrist. The typical clinical manifestation is that of a slow-growing, poorly circumscribed soft tissue mass that does not limit joint motion [48]. Radiography and ultrasound may show a nonspecific soft tissue mass with a variable extent of fine, stippled calcifications (Fig. 14) [48]. CT is optimal for depicting the calcified areas of the lesion, with other regions demonstrating nonspecific soft tissue attenuation [48]. At MRI, calcifying aponeurotic fibroma appears as a round to oval superficial subcutaneous soft tissue mass with an ill-defined appearance, and a tendency to infiltrate into or adhere to the surrounding tissues [48–50]. Masses usually show low to isointense T1 signal intensity, overall heterogeneously high T2 signal intensity (Fig. 14), and heterogeneous intense enhancement [40, 50]. There are no reliable imaging features which discriminate calcifying aponeurotic fibroma from soft tissue sarcoma (particularly synovial sarcoma and undifferentiated pleomorphic sarcoma) [48, 50]. Therefore, biopsy and pathological analysis are warranted.

## Calcific myonecrosis

**Fig. 15:**
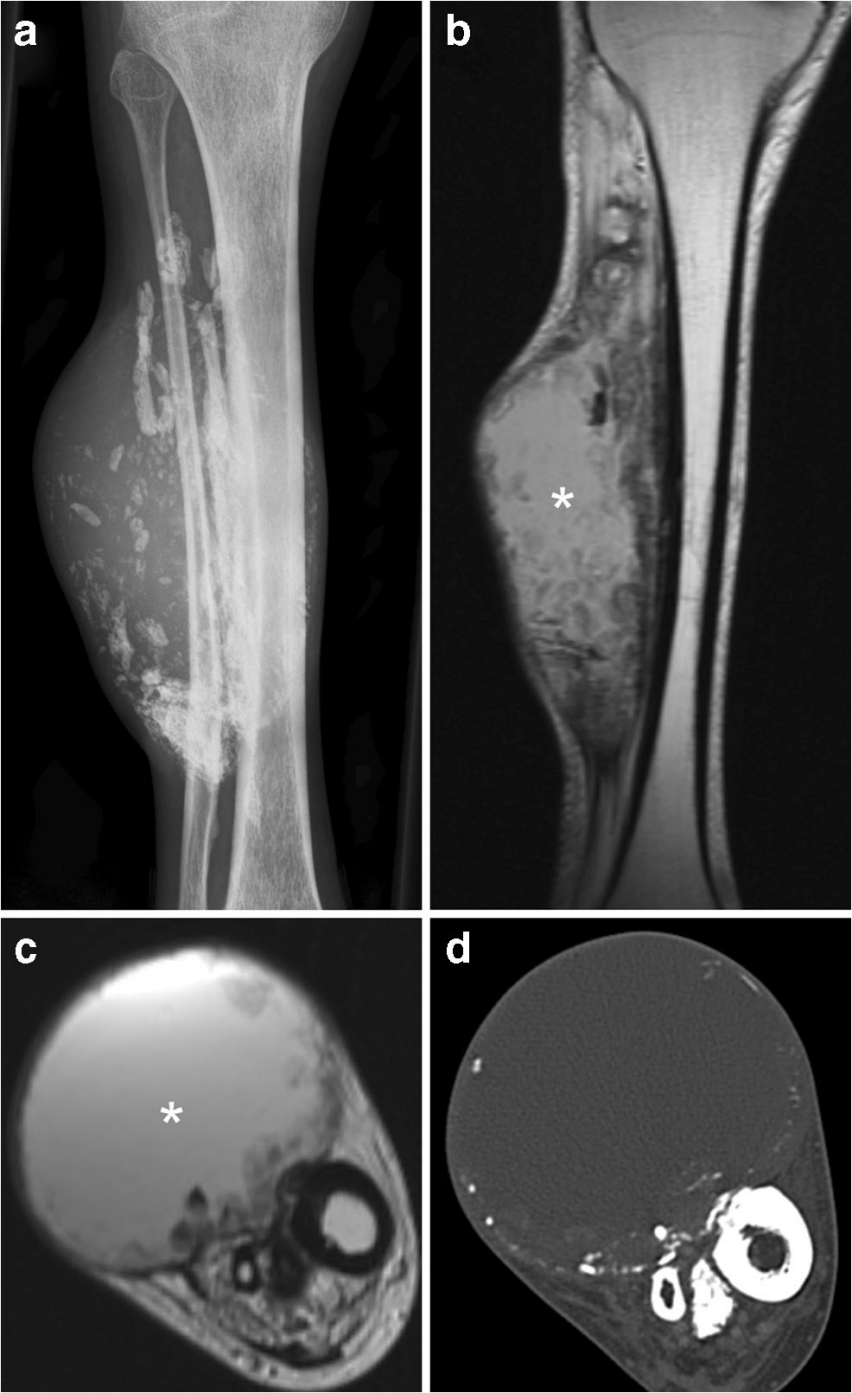
A 72-year-old man with calcific myonecrosis of the anterior compartment of the right leg. **a** Radiography shows a soft tissue mass with extensive calcifications. **b, c** Axial (**a**) and coronal (**b**) proton-density weighed images shows the mass to be located within the anterior compartment of the right lower leg, with a liquid-appearing center. d Axial CT shows the typical peripheral calcifications

Calcific myonecrosis is a rare posttraumatic entity, in which a muscle is replaced by a fusiform mass with central liquefaction and peripheral calcifications [51, 52]. It is assumed that calcific myonecrosis results from muscle ischemia and cystic degeneration, which is supported by the strong association with a remote history of compartment syndrome and/or vascular injury after fracture [51, 52]. Patients often present many (reportedly 10 to 64) years after the inflicting traumatic event [51, 52]. Characteristic symptoms are a slowly enlarging, usually painful soft tissue mass in the anterior compartment of the lower leg [51, 52]. Enlargement of the mass is caused by repeated intralesional hemorrhage [51]. Although less common, calcific myonecrosis can also occur in other compartments of the lower leg, the thigh, and forearm [51]. The long time interval after the inflicting trauma and presence of alarming clinical symptoms may lead to suspicion of malignancy or infection. Calcific myonecrosis has a typical imaging appearance. Radiography shows a mass with peripherally oriented plaque-like amorphous calcifications (Fig. 15) [51, 52]. These calcifications are present within the entire involved muscle and usually linear in orientation and sheet-like [51, 52]. Smooth bony erosions may be present (presumably due to a chronic pressure effect), with minimal periosteal reaction [51, 52]. CT and MRI readily show the compartmental involvement of a well-defined mass with a liquid-appearing center and peripheral calcifications (Fig. 15) [51, 52]. Fluid-calcium levels may be seen representing communication between the necrotic muscle and the tendon sheath [52]. Calcific myonecrosis typically shows a homogeneous intermediate T1 signal intensity and a heterogeneously high T2 signal intensity throughout the central liquid-appearing region, and no enhancement [52].

## Ancient schwannoma

**Fig. 16.**
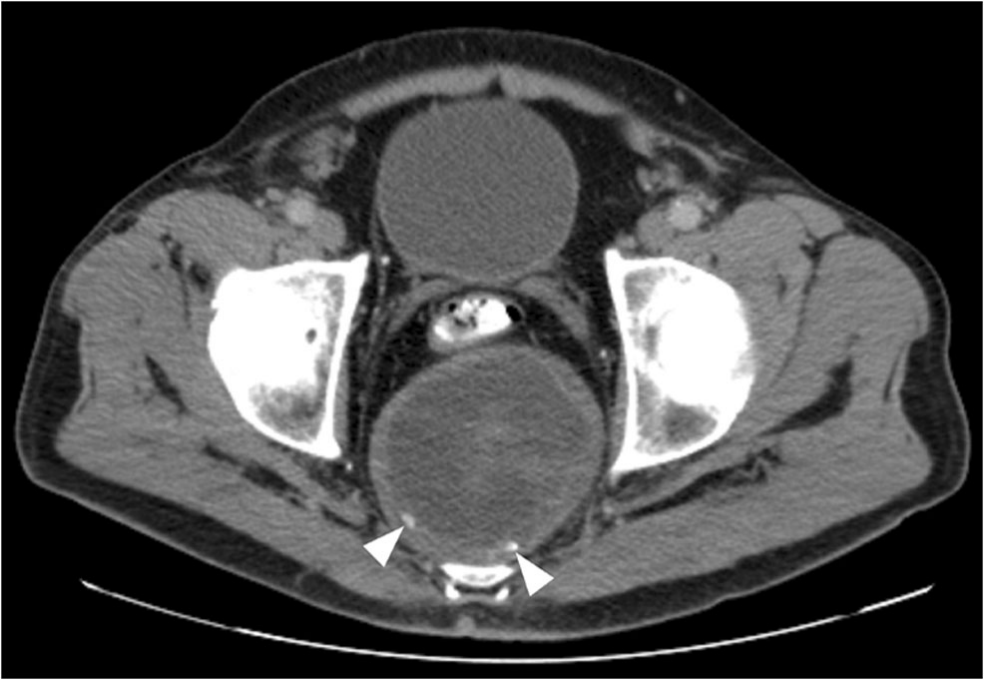
A 69-year-old man with ancient schwannoma in the presacral space. Axial CT shows a well-defined mass with hypodense center, enhancing capsule, and peripheral calcifications (*arrowheads*) in the presacral space. Pathological analysis after biopsy confirmed ancient cystic schwannoma. The mass remained unchanged after 10 years’ follow-up

Ancient schwannomas are long-standing, slow-growing schwannomas that show advanced degeneration. They are usually located deep in the head and neck, chest, retroperitoneum, pelvis, and extremities of elderly patients [53]. Because of their deep location, they can grow considerably large before becoming symptomatic. Ancient schwannomas typically show cystic necrosis and calcifications [53–55], findings that can be identified on imaging (Fig. 16). Calcification is the usual degenerative change, but ossification may also occur although rarely [53]. Because ancient schwannoma can contain cystic necrotic areas and hemorrhage, they may mimic sarcoma or malignant peripheral nerve sheath tumor [53–55]. It has been suggested that the presence of an enhancing fibrous capsule and the “split fat sign” (a rim of fat surrounding the tumor) at MRI are helpful to differentiate ancient schwannoma from malignant tumors [55]. However, the “split fat sign” is only seen in ancient schwannomas in the extremities and it does not exclude malignant peripheral nerve sheath tumor. In addition, the “split fat sign” is non-specific and can be seen in other entities besides neurogenic tumors. Furthermore, peripheral enhancement may also be seen in malignant tumors. Therefore, biopsy usually is indicated to exclude malignancy.

## Castleman disease

**Fig. 17:**
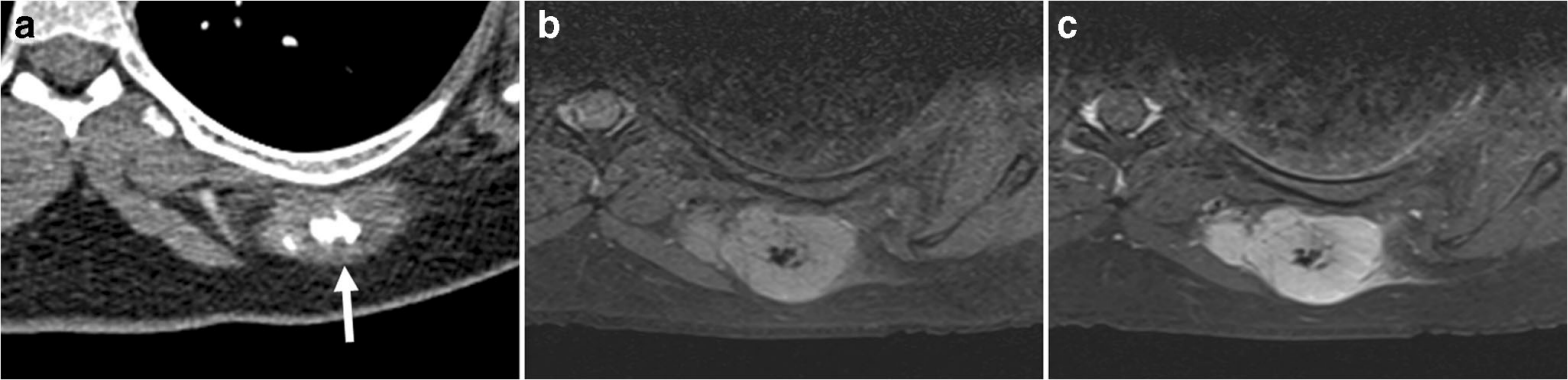
A 22-year-old woman with Castleman disease. **a** Axial CT shows a well-defined subcutaneous soft tissue mass with central calcification at the left thoracic region. **b, c** Axial pre- (**b**) and postgadolinium (**c**) T1-weighted images with fat suppression show homogeneous enhancement of the mass (except for the central calcification). The mass was biopsied and pathologically proven to be Castleman disease

Castleman disease is an uncommon benign nonclonal lymphoproliferative disorder, which may present as a localized or multicentric form. The localized form typically occurs earlier in life (3rd and 4th decades) and may be asymptomatic. The multicentric form occurs later in life (5th and 6th decades) and systemic inflammatory symptoms are common [56, 57]. Although Castleman disease primarily involves lymphatic tissues, any extralymphatic site may be involved [57, 58]. Therefore, it can present as a worrisome soft tissue lesion. The typical imaging appearance is that of a solitary enlarged lymph node or localized nodular lesions which demonstrate homogeneous and intense enhancement, both at CT and MRI [57–59]. Lesions tend to be heterogeneously T1 iso- to hyperintense relative to muscle and T2 hyperintense [57–59]. Importantly, approximately 5–10% of lesions have internal calcifications at CT (Fig. 17) [57, 58], a feature which helps to differentiate them from untreated lymphoma [59]. Biopsy is needed to confirm the diagnosis and to perform subtype classification [56].

## Summary

The typical clinical features, specific imaging features, and main differential diagnostic considerations of calcified or ossified benign soft tissue lesions that may simulate malignancy are summarized in Table 1. The diagnosis of a benign vascular lesion, calcific tendinopathy with osseous involvement, Hoffa’s disease, tumoral calcinosis, and calcific myonecrosis can usually be confidently established by their typical clinical presentations, locations, and imaging characteristics. Some entities, however, can be more diagnostically challenging. Diagnosis of myositis ossificans can be difficult in the early and intermediate stages, and biopsy or close imaging surveillance are warranted in indeterminate cases. Tophaceous gout with atypical presentation can closely resemble malignancy such as synovial sarcoma. Biopsy or dual-energy CT can establish the definite diagnosis. Periosteal chondroma and periosteal chondrosarcoma may only be distinguished on imaging by size (lesions > 3 cm are more likely chondrosarcoma). Primary chondromatosis may be very difficult to distinguish from malignant transformation to synovial chondrosarcoma, both pathologically and radiologically. Although malignant transformation is uncommon, multiple recurrences and development of marrow invasion should be considered alarming features. Osteolipomas may show intense and heterogeneous enhancement and resemble soft tissue sarcoma. There are no definite imaging features either which reliably discriminate calcifying aponeurotic fibroma and ancient schwannoma from malignancy. In these cases, biopsy and pathological analysis are warranted.

**Table 1:** Typical clinical features, specific imaging features, and main differential diagnostic considerations of calcified or benign soft tissue lesions that may simulate malignancy

Calcified or ossified benign soft tissue lesion	Typical clinical features	Specific imaging features	Main differential diagnostic consideration(s)
Myositis ossificans	Pain, swelling, and joint stiffness following blunt soft tissue trauma	Calcified peripheral rim with a lucent center (end of intermediate stage) and pronounced peripheral bone formation (mature stage)	Soft tissue osteosarcoma
Tophaceous gout	Frequency increases with age, male predominance, elevated serum urate levels	Multifocality and presence of monosodium urate crystals, which can accurately be identified by dual-energy CT	Soft tissue sarcoma (synovial sarcoma)
Soft tissue venous malformation	Superficial lesions appear as faint blue, soft, easily compressible, and nonpulsatile masses, which enlarge with Valsalva maneuver	Presence of phleboliths, lack of flow voids, and slow gradual enhancement (of note, high flow vascular lesions tend to produce more “tram-track” calcifications seen in arteries)	Other soft tissue vascular malformations (e.g., lymphatic or arteriovenous malformation)
Calcific tendinopathy with osseous involvement	Pain and limitation of range of motion around the affected area	Location of calcifications in a tendon, absence of an associated soft tissue mass, and prominent associated bone marrow edema	Malignancy (e.g., soft tissue sarcoma, periosteal sarcoma, or metastatic disease) or infection
Periosteal chondroma	Often asymptomatic, but may present as palpable and painful masses with mechanical symptoms	Calcified chondroid matrix with scalloping and overhanging edges of cortex of adjacent bone and size < 3 cm	Bizarre parosteal osteochondromatous proliferation, periosteal chondrosarcoma
Primary synovial chondromatosis	Knee and hip are predilection sites	Innumerable and similarly shaped intraarticular calcifications, chondroid ring-and-arc pattern of mineralization	Malignant transformation to synovial chondrosarcoma
Hoffa’s disease	In young women and persons practicing jumping sports	Low T1 and T2 signal intensity areas representing fibrin and hemosiderin (subacute and chronic phases), and presence of ossified fragments (chronic phase)	Synovial sarcoma, venous malformation
Tumoral calcinosis	Painless mass(es) around the hip, elbow, shoulder, foot, or wrist	Lobulated calcific and cystic soft tissue mass, multifocality	Calcinosis associated with chronic renal failure*
Lipoma with metaplastic bone	Symptoms due to mass effect	Presence of fat, bone, and calcifications	Soft tissue sarcoma (liposarcoma)
Calcifying aponeurotic fibroma	Slow-growing mass that does not limit joint motion in children and adolescents (males more commonly than females)	Most commonly affects the deep volar fascia, tendons, and aponeuroses of the hands, followed by the feet, and may calcify	Soft tissue sarcoma (e.g., synovial sarcoma or undifferentiated pleomorphic sarcoma)
Calcific myonecrosis	Slowly enlarging, usually painful mass in the anterior lower leg compartment, and remote history of compartment syndrome and/or vascular injury after fracture	Compartmental involvement of a well-defined mass with liquid-appearing center and peripheral calcifications	Soft tissue sarcoma, infection
Ancient schwannoma	In elderly patients, located deep in the head and neck, chest, retroperitoneum, or pelvis	Cystic necrosis and calcifications	Soft tissue sarcoma, malignant peripheral nerve sheath tumor
Castleman disease	Either asymptomatic or local symptoms due to mass effect in unicentric disease, whereas B-symptoms, generalized lymphadenopathy, hepatomegaly, and splenomegaly can be seen in multicentric disease	Internal calcification (in 5–10% of lesions) and homogeneous and intense enhancement	Lymphoma, soft tissue sarcoma

*Tumoral calcinosis and calcinosis associated with chronic renal failure have similar radiologic and histopathologic appearance. Therefore, whenever the diagnosis of tumoral calcinosis is suggested by imaging, calcium-phosphate metabolic parameters and renal function should be checked

## Conclusions

There is a wide spectrum of calcified or ossified benign soft tissue lesions that may simulate malignancy. Knowledge of their clinical presentations, locations, and imaging characteristics is essential to make a correct diagnosis or to narrow the differential diagnosis, and to recommend justified further imaging or tissue sampling when a certain diagnosis in terms of benignancy or malignancy cannot be made.
